# Animals evoking fear in the Cradle of Humankind: snakes, scorpions, and large carnivores

**DOI:** 10.1007/s00114-023-01859-4

**Published:** 2023-07-05

**Authors:** Daniel Frynta, Hassan Sh Abdirahman Elmi, Kateřina Rexová, Markéta Janovcová, Veronika Rudolfová, Iveta Štolhoferová, David Král, David Sommer, Daniel Alex Berti, Petra Frýdlová

**Affiliations:** 1 Department of Zoology, Faculty of Science, Charles University, Viničná 7, 128 43 Prague 2, Czech Republic; 2 Amoud University, Borama, Somaliland

**Keywords:** Fear, Evolutionary psychology, African savanna, Non-WEIRD, Ophidiophobia, Arachnophobia

## Abstract

Theories explain the presence of fears and specific phobias elicited by animals in contemporary WEIRD (Western, educated, industrialized, rich, and democratic) populations by their evolutionary past in Africa. Nevertheless, empirical data about fears of animals in the Cradle of Humankind are still fragmentary. To fill this gap, we examined which local animals are perceived as the most frightening by Somali people, who inhabit a markedly similar environment and the region where humans have evolved. We asked 236 raters to rank 42 stimuli according to their elicited fear. The stimuli were standardized pictures of species representing the local fauna. The results showed that the most frightening animals were snakes, scorpions, the centipede, and large carnivores (cheetahs and hyenas). These were followed up by lizards and spiders. Unlike in Europe, spiders represent less salient stimuli than scorpions for Somali respondents in this study. This conforms to the hypothesis suggesting that fear of spiders was extended or redirected from other chelicerates.

**Supplementary Information:**

The online version contains supplementary material available at 10.1007/s00114-023-01859-4.

## Introduction

Many studies focusing on fear evoked by animal stimuli were performed in WEIRD populations (e.g., Polák et al. 2020; Staňková et al. 2021). Although animals represent almost no real risk for people from these populations, fear of certain species or taxa is widespread, even in a non-clinical population. Moreover, uncontrolled fear of animals may result in specific phobias, which belong to the most common psychiatric diagnoses (Fredrikson et al. 1996; Coelho et al. 2020; Polák et al. 2016, 2022, and references herein). Snakes, spiders, and large carnivores were repeatedly mentioned as triggers in both of these contexts (see below). The presence of high fear of or even phobias in the absence of reliable actual threat has led to explanations suggesting that sensitivity for specific animal stimuli has evolved in the evolutionary past, either in our deeper primate ancestors (Isbell 2006, 2009) or in the hominid lineage during the late Miocene-Pleistocene period (Coelho and Purkis 2009).

The snake represents an extremely salient fear stimulus not only to humans (e.g., Öhman and Mineka 2001; Prokop et al. 2018; Rádlová et al. 2019) but also to other primates (e.g., Weiss et al. 2015; Isbell and Etting 2017). Many authors have proposed that the human brain is deeply adapted to respond to snake stimuli (Isbell 2006). Phenomena such as biological preparedness (Seligman 1971; Öhman and Mineka 2001), prioritization of stimuli (Fabio and Caprì 2019), attentional bias (Öhman et al. 2001, 2012), and specific neural mechanisms (Van Strien and Isbell 2017; Van Le et al. 2013; Bertels et al. 2023) were repeatedly studied in this context (for a review of the experimental evidence, see Baynes-Rock 2017).

Spiders are distinct and salient stimuli (Landová et al. 2021), which were considered biologically relevant. Nevertheless, the vast majority of spiders are venomous, but only a handful of them may represent a threat to humans (Hauke and Herzig 2017). Moreover, most of these highly venomous spiders are distributed in Australia and the USA, clearly outside the distribution range of human ancestors (see Frynta et al. 2021). In contrast, scorpions, which are related to spiders (both are chelicerates), are more venomous and abundant in the geographic range of human ancestors. Thus, the fear of spiders might be a redirected or extended fear of scorpions (Vetter et al. 2018; Frynta et al. 2021; Rudolfová et al. 2022).

Mammalian megafauna most likely represented a threat to early hunter-gatherers during our evolutionary past. However, the distribution range and abundance of these animals have been dramatically reduced during the last centuries. Despite this, many big mammals, in particular the large carnivores, elicit negative emotions in the contemporary world (Kansky et al. 2014; Dressel et al. 2015; Johansson et al. 2016).

Regardless of the crucial role of evolutionary experience in the theories explaining the fear response of contemporary humans to animal species, empirical studies of fear evoked by animal stimuli in native human populations inhabiting the ancestral environment are still scarce (but see Kaltenborn et al. 2006; Prokop et al. 2010; Onyishi et al. 2021). There is a strong consensus among scholars that hominins including modern *Homo sapiens sapiens* have evolved in African savannas (Stringer 2016; Dawkins and Wong 2016). We focus on the African Horn, the region where the continuous presence of human ancestors for the last several millions of years has been clearly documented (e.g., McDougall 2005; Profico et al. 2016; White et al. 2003). Currently, genetic data uncovered complex intra-African migration admixtures prior to the further spread of anatomically modern humans out of Africa (Rito et al. 2019). Anyway, the African Horn belongs to the source areas for colonization of the Arabian Peninsula and Eurasia via the Bab al-Mandab, which is the likely staging point for out-of-African dispersals (Groucutt et al. 2015).

Moreover, contemporary WEIRD societies are affected by intensive urbanization and loss of experiences with nature, which can affect their fear response to animal stimuli (e.g., Soga and Gaston 2016). Thus, there is an urgent call for studies performed in more traditional societies including the African ones.

In this study, we examined self-reported fear evoked in Somali respondents by 42 animal species represented by picture stimuli. We hypothesized that (1) the most frightening are common species representing a real threat to local people, i.e., venomous snakes, scorpions, and large mammals such as spotted hyenas and Hamadryas baboons. (2) Spiders evoke less fear than scorpions.

## Materials and methods

### The respondents

The research was performed at the campus of Amoud University in Borama. Most of the subjects were undergraduate students in various fields who agreed to voluntarily participate in the experiment. Owing to the importance of Amoud University, the students came not only from the Borama region itself but also from other provinces of Somaliland and adjacent Somali-speaking countries. A total of 236 Somali respondents performed the task (for the data, see Online resource 1). They were 214 men and 22 women. The mean age was 21.83 years (median = 22, range 18–35, see Online resource 2 for age structure). The sex ratio of respondents was strongly male-biased, as women were underrepresented among students at Amoud University, and moreover, they agreed to participate in the study less frequently (see under the Limitations of the study).

### The stimuli

We selected 42 species belonging to the local fauna of Somaliland and adjacent areas. We tried to include representatives of all principal groups of land vertebrates, ten species of mammals (aardvark, yellow-spotted rock hyrax, crested porcupine, hamadryas baboon, cheetah, spotted hyena, black-backed jackal, desert warthog, East African oryx, and Speke’s gazelle), ten birds (Somali ostrich, vulturine guineafowl, ring-necked dove, white-bellied go-away-bird, kori bustard, marabou stork, Rüppell’s vulture, secretarybird, black-winged kite, and superb starling), four lizards (Taylor’s fat-tail gecko, white-throated savanna monitor, Vaillant’s strange agama, and ocellated uromastyx), four snakes (African house snake, red spitting cobra, Arabian cat snake, and Egyptian saw-scaled viper), two chelonians (Somalian helmeted terrapin and leopard tortoise), and three frogs (Blanford’s toad, Anchieta’s ridged frog, and Kachowski’s sand frog). In addition, we included the most relevant invertebrates. These were three scorpions (burrowing scorpion, thick-tailed scorpion, and nomad scorpion), two spiders (stone huntsman spider and Cameroon red baboon tarantula), one camel spider, two insects (migratory locust and Somali flower chaffer beetle), and one centipede (for a list and scientific names of the stimuli, see Online resource 3).

### Stimuli preparation

For each species on the list, we selected a relevant picture. The source photographs of invertebrates, amphibians, and reptiles were adopted from the authors’ archives and the archives of Tomáš Mazuch; birds and mammals were from respectable monographs (del Hoyo et al. 1996-2003; Castelló 2016; Wilson and Mittermeier 2009-2014). Four species were from online sources (see Online resource 3). To avoid possible effects of the background and size of the stimulus on ratings, we digitally placed the animals on a white background. We also resized them so that the pictured animals were of similar size. Then, we printed the final stimuli as photographs, 130 × 90 mm in size. We previously showed that self-reported fear of standardized pictures highly correlates with that of live animals (Landová et al. 2012).

### The task

At the beginning of the task, a respondent was standing in front of a well-lit table. We provided him/her with a set of 42 pictures packed in random order. We asked the respondent to imagine the pictures as real animals. Then we asked him/her to place all stimuli on the table in a random assemblage. This sometimes required assistance to ensure that the stimuli are oriented properly, i.e., the top margins of the stimuli were oriented towards the top of the table. The task was to pick up the picture of an animal that was the most fear-evoking, then pick up the second most fear-evoking one, until he/she picked up the least fear-evoking stimulus on the table. In the end, the respondent had a whole pack of pictures in his/her hand. We asked the respondent to have a look at all stimuli in each round. The picture order in the pack was then coded from 1 (the most fearful one) to 42 (the least fearful one), further referred to as ranks.

We previously applied this rank-order method in multiple studies evaluating either the beauty of animal stimuli (e.g., Marešová et al. 2009a, b; Frynta et al. 2011, 2013; Lišková and Frynta 2013; Landová et al. 2018b) or emotions evoked by animals (e.g., Rádlová et al. 2019). It maximizes the informative content of the respondents’ judgment by covering the full ordination scale (Lišková et al. 2015). We repeatedly demonstrated that mean ranks were highly correlated to scores produced by the 5- or 7-point Likert scale (e.g., Frynta et al. 2010; Rádlová et al. 2020).

The study was approved by the Institutional Review Board of Charles University, Faculty of Science, approval no. 2019/2011, granted on 27 March 2019.

### Data analysis

The data were ranked; thus, we adopted methods appropriate for this type of dataset, primarily non-parametric statistics. To quantify agreement among the respondents, we computed Kendall’s coefficient *W* as implemented in the package irr (Gamer et al. 2022). To compare the mean ranks of individual stimuli, we first computed the Friedman test, enabling us to prove the significant effect of species. Then, we employed the post hoc Friedman-Neményi test, permitting reliable multiple comparisons among the stimuli. The output was a matrix of *P*-values. These tests are available in the PMCME and PMCMRplus packages (Pohlert 2014). These calculations were carried out in the R-environment (R Development Core Team 2010). Means and median values of ranks were computed for each stimulus/species and further analyzed. To obtain more intuitive values increasing with fear (not decreasing as original ranks and its means) and ranging from 0 to 100, we calculated the following index: fear = 100 − (100 * (median rank − 1) / (the number of examined stimuli − 1)).

Cluster analysis was employed to uncover groups of stimuli treated by the respondents in a correlated way. The dissimilarity matrix was extracted from the ranking dataset (1-Pearson’s *r*) and applied to Ward’s method of clustering. The calculations were performed in Statistica 9.1 (Statsoft Inc. 2010).

## Results

We found considerable agreement among 236 Somali raters (Kendall’s coefficient of concordance, *W* = 0.634). Basic descriptive statistics for each of the 42 stimuli (mean rank, median, quartiles, and standard deviation) can be found in Online resource 3. Friedman’s test proved that the effect of the stimulus on perceived fear was highly significant (chi-square = 6130.252, df = 41, *P* < 0.0001). Out of the 861 post hoc comparisons among stimuli (= species), 654 (76%) were significant (*P* < 0.05, for the matrix of *P*-values, see Online resource 4).

**Fig. 1:**
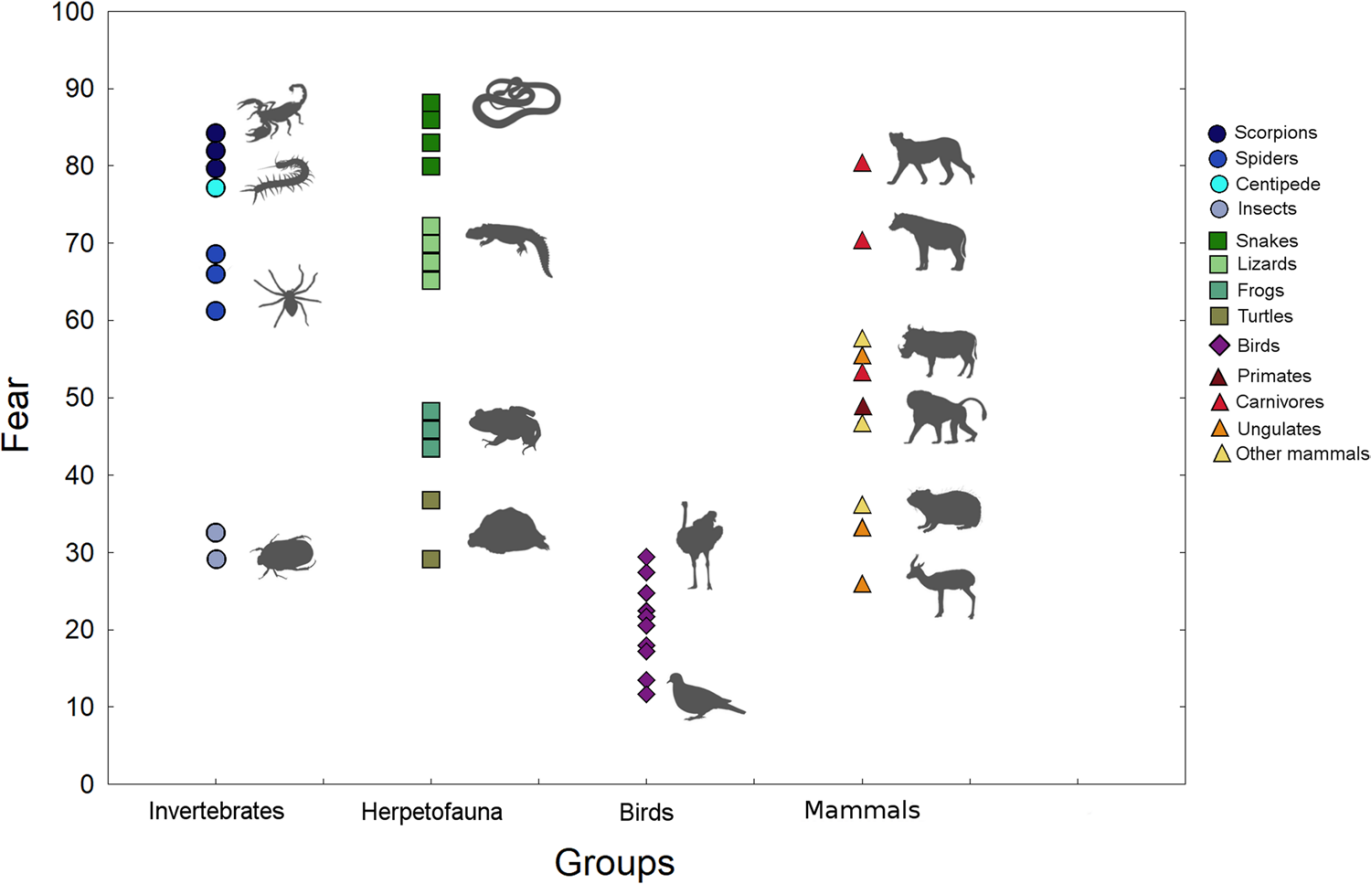
Pattern of fear elicited by 42 animal stimuli in 236 Somali raters. The stimuli are arranged according to fear (vertical axis, higher the score, greater the fear) and divided into four distinct life forms. Thirteen specific taxa are marked by symbols (see the legend). For calculation of the fear from the mean ranks, see the “Materials and methods” section

The distribution pattern of self-reported fear among the animal taxa is visualized in Fig. 1. The stimuli ranked by the Somali raters as the most frightening (Online resource 3) were snakes ((1) Egyptian saw-scaled viper, (2) African house snake, (3) red spitting cobra, and (6) Arabian cat snake positions), scorpions ((4) burrowing scorpion, (7) thick-tailed scorpion, and (8) nomad scorpion), large carnivores ((5) cheetah and (11) spotted hyena) and (9) centipede. These are followed by lizards ((10) ocellated uromastyx, (12) white-throated savanna monitor, (13) Vaillant’s strange agama, and (14) Taylor’s fat-tail gecko) and spiders ((15) Cameroon red baboon tarantula, (16) camel spider, and (17) spider *Eusparassus walckenaeri*).

The top eight frightening stimuli (snakes, cheetahs, and scorpions) form a homogenous group according to post hoc comparisons (*P* > 0.05). The hyena was significantly less frightening than the top four stimuli (snakes and one scorpion), but not the cheetah. Similarly, all lizards were significantly less frightening than the top three stimuli (snakes). All comparisons between “spiders” (including the camel spider) and the top eight frightening stimuli (snakes, cheetahs, and scorpions) were significant (for details, see Online resource 4).

The other stimuli were significantly less frightening than the abovementioned 16 ones, i.e., snakes, scorpions, the centipede, large carnivores, lizards, and spiders. The birds were placed consistently as the least frightening stimuli, followed by turtles and insects. The other mammals and frogs showed intermediate values.

**Fig. 2:**
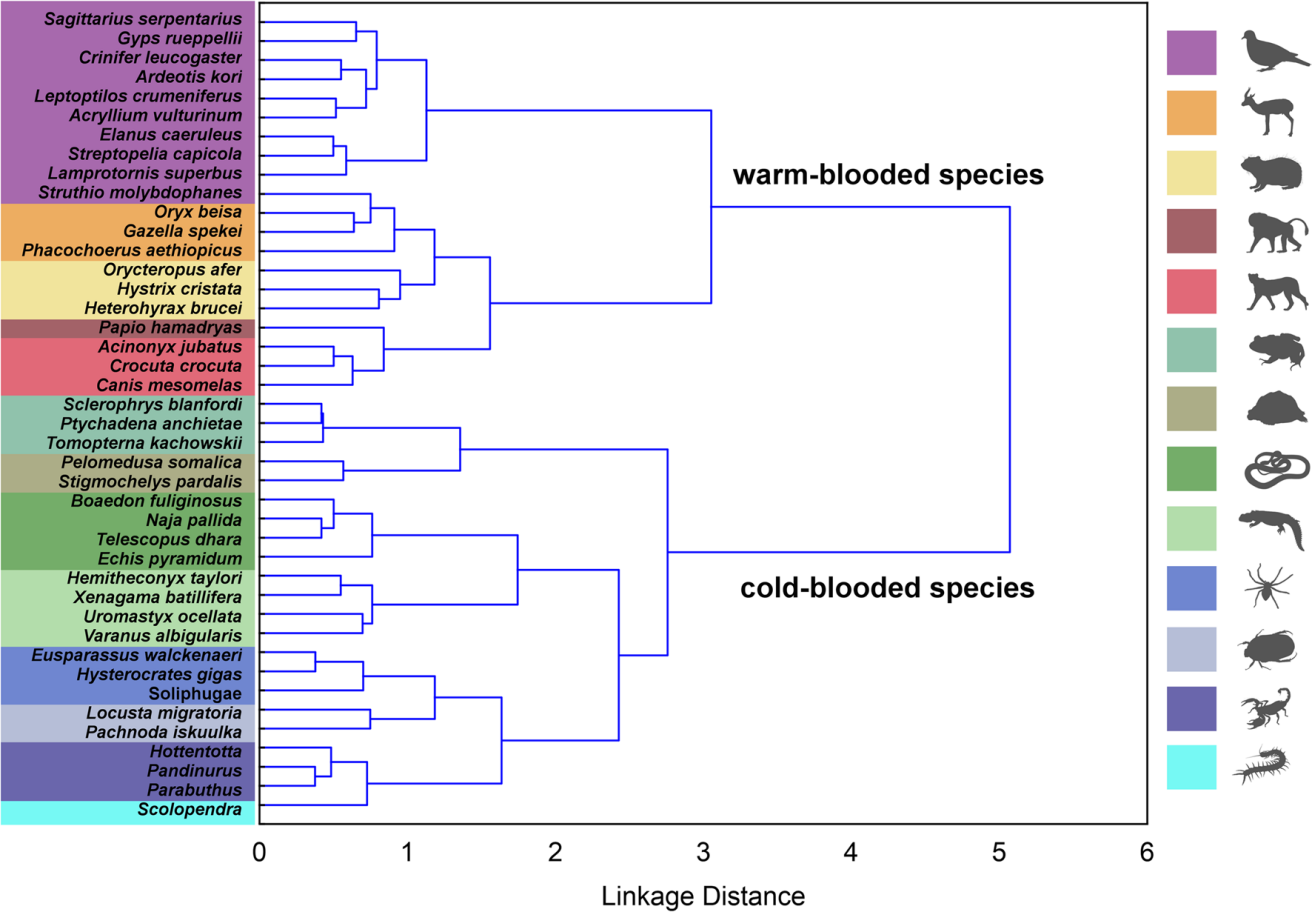
Cluster analysis of 42 animal stimuli according to fear ranking performed by 236 Somali raters. It extracts and visualizes the hidden pattern of unsupervised categorization in the dataset. The dissimilarity matrix is based on Pearson’s correlations (1-*r*) among ranks. We adopted Ward’s method of clustering

Cluster analysis of the dataset revealed a clear structure corresponding to higher taxa of animals (Fig. 2). There was a main division between warm-blooded and cold-blooded animals. The former branch further splits into clusters of flying birds and mammals (including the ostrich). The latter branch splits into clusters of frogs/turtles, squamates (snakes and lizards), and invertebrates (scorpions + the centipede and insects + spiders).

## Discussion

We demonstrated that snakes, scorpions, large carnivores, and the centipede are perceived as the most frightening animals by Somali respondents in this study. This fear seems to be reasonable as these animals may cause injuries or even the death of humans.

A strong correspondence between the clustering of the stimuli and the higher taxa and/or morphotype to which the stimuli species belong (Fig. 2) suggests that the fear ranking performed by the Somali raters included a considerable categorization component. The stimuli belonging to a certain cognitive category received a similar evaluation by a given rater. This makes it reasonable to further discuss the results not only in terms of individual stimuli (species) but also in terms of these categories.

### Venomous animals

Some species of snakes (Casewell et al. 2020), scorpions (Isbister and Bawaskar 2014), and centipedes (Undheim and Jenner 2021; Mavridis et al. 2016) possess potent toxins able to envenomate mammals including humans and other primates (Chippaux 2006; Chippaux and Goyfon 2008; Valenta 2010; Ombati et al. 2018). Stimuli belonging to these groups including highly venomous species occupy 7 of the top 8 stimuli eliciting the highest fear in our Somali respondents. Thus, fear of these animals can be interpreted as a simple utilitarian response to this threat. Current phylogenetic studies showed that the toxicity of certain lineages of venomous snakes (Harris et al. 2021), as well as scorpions of the family Buthidae (Sanitbanez-Lopez et al. 2020), has co-evolved with their mammalian predators, and thus, their toxins are adjusted to act on mammals.

Our set of stimuli includes both deadly venomous (Egyptian saw-scaled viper and red spitting cobra) and non-venomous (African house snake) or only slightly venomous (Arabian cat snake) snakes. All of them appeared to be top frightening among our stimuli. Therefore, the fear response to a snake was not specific to venomous species. This conclusion is, however, confined to a wide-scale comparison. Studies enabling finer comparisons among multiple snake species regularly report a clear association between the stimulus morphotype and the elicited fear response (Landová et al. 2018a; Rádlová et al. 2019).

Similarly, fear elicited by three scorpion stimuli was mutually comparable, although the consequences of envenomation by these genera differ sharply (Forde et al. 2022). The thick-tailed scorpions (*Parabuthus*) have highly potent venom (Isbister and Bawaskar 2014; Schaffrath et al. 2018; Kovařík et al. 2019; Ward et al. 2018), while the burrowing scorpions, despite their large body size, represent no reliable danger to humans. Thus, also in this case, fear of scorpions is a response to the category of stimuli rather than to certain species.

### Large carnivores and megafauna

Africa is the homeland of many mammalian species, which are able to seriously wound or even kill humans (Treves and Naughton-Treves 1999). Cheetahs and spotted hyenas belonged to the animal stimuli eliciting the highest fear in our Somali respondents. The cheetah specializes in relatively small prey such as gazelles; attacks on humans are thus almost absent (Mills and Mills 2017). Therefore, the position of the cheetah among the most frightening animals is a bit surprising. The respondents may have confused cheetah with leopard (*Panthera pardus*), preferring prey within the range of 10–40 kg (Hayward et al. 2006). This large cat can be really dangerous (for early reports from Somaliland, see Elliot 1897).

In Somaliland, the spotted hyena is a very common species (Evangelista et al. 2018). On the Amoud University campus, we recorded regular whoop calls from at least three packs and found multiple dens nearby. Reports from the Tigray Province in Ethiopia proved that the spotted hyaenas regularly consume human remains and occasionally may attack or even kill people (Abay et al. 2011; Yirga et al. 2015). In our dataset, the spotted hyena belongs to the highly frightening animals (11th out of 42 examined stimuli); however, it was significantly less frightening than three snake and one scorpion stimuli. A study performed in the Serengeti Region of Tanzania reported that the spotted hyena elicited less fear than the cheetah or snake (Kaltenborn et al. 2006). In Kenyan agropastoralist communities, it is perceived as ugly, and the attitude toward this species is negative (de Pinho et al. 2014).

In baboon societies, especially the Hamadryas baboon, aggression is frequent (Evans et al. 2022). Thus, these primates possessing large canines are perceived as potentially dangerous for humans by villagers in many African regions (Uganda: Hill 1997). In spite of this, our respondents evaluated the fear elicited by the baboon only by median ranks (21st position).

From a historical perspective, additional megafauna potentially dangerous for humans was present in the Somaliland territory until recently; this especially concerns lions (Gebresenbet et al. 2018; Packer et al. 2005, 2011; Matema and Andersson 2015) and elephants (Dunham et al. 2010). These animals have gone extinct in this region and thus were not included in our set of stimuli. The absence of permanent rivers and larger freshwater pools in Somaliland and adjacent areas precludes the presence of buffaloes, hippopotamuses, and crocodiles, the animals responsible for human casualties in other parts of Africa (Kanga et al. 2012; Chomba et al. 2012; Dunham et al. 2010).

In the Serengeti, more local people reported strong fears of lions, leopards, elephants, buffalos, crocodiles, and hippopotamuses than of any species represented in our set of stimuli (Kaltenborn et al. 2006). This may explain why the positions of the top frightening animals in our results were occupied predominantly by cold-blooded animals like snakes and scorpions instead of mammalian megafauna.

### Other frightening animals

Somali respondents rated both lizards and spiders as significantly more fear-eliciting than 25 of the 42 examined stimuli, i.e., mammals (except cheetahs and hyenas), frogs, turtles, and birds. Although most lizards (in Africa except for the monitor lizards) are harmless animals representing no real danger to humans, some of them are believed to be venomous in many countries including certain parts of Africa, the Middle East, and even Southern Europe (Ceríaco et al. 2011; Ceríaco 2012). This irrational belief could explain their relatively high fear rating detected in this study. It was demonstrated that attitudes towards particular animals can be a subject of social transmission in childhood (Reider et al. 2022). Nevertheless, lizards were still considerably less frightening stimuli than snakes. This fairly corresponds to findings from Central Europe suggesting that snakes consistently elicit more fear than lizards (Janovcová et al. 2019).

The spider stimuli were rated by Somalis as much less frightening than the scorpions. A study from Central Europe reported no such difference (Frynta et al. 2021; Staňková et al. 2021). This is in agreement with our previous finding that the Somali people, in contrast to the Europeans, exhibit a strong attentional bias towards scorpions when presented simultaneously with spiders on the screen (Rudolfová et al. 2022). In the USA, self-reported fear scores were either equal to or significantly higher for scorpions than spiders; however, the presence of scorpions in the study area had no effect on these results (Vetter et al. 2018). This may suggest that actual exposure to venomous scorpions in the environment is not sufficient to change levels of specific fears. Thus, the fear pattern we recorded in Somaliland may be attributed to long-term rather than short-term effects.

We showed that in the ancestral environment, spiders are perceived as relatively less frightening stimuli than in Europe. This is consistent with the view that fear of spiders is an extension of more biologically relevant fear induced by other chelicerates, in particular, scorpions (Vetter et al. 2018; Frynta et al. 2021). Nevertheless, even on the African continent, spiders are still perceived as frightening stimuli, and the fear of spiders belongs to the common fears (Muris et al. 2008).

### Limitations of the study

(1) We collected data about self-reported (explicit) fear, but evolutionary interpretations deal with implicit fear that was not directly measured in this study. Nonetheless, these two are not completely independent. There is some evidence that self-reported fear of animals correlates with physiological response such as skin resistance (snakes: *r* = 0.77, Landová et al. 2020) or fMRI activation (Landová et al. 2023). Specifically in Somaliland, supportive evidence comes from eye-tracking studies focusing on scorpions (Rudolfová et al. 2022) and snakes (Štolhoferová et al. submitted). Moreover, modified behavioral reaction, not the implicit fear per se, is the ultimate base for adaptive selection, and explicit fear was also shown to correlate with altered behavior (behavioral approach test—Koch et al. 2002; virtual reality—Kisker et al. 2021). Nevertheless, a cross-cultural comparison of, e.g., physiological responses to various animal stimuli is of great interest to verify the interpretation of our results. (2) The age and gender composition of our sample are biased in favor of young men. This limits generalizing our results. Women are typically more sensitive to animal stimuli and usually report higher levels of fear (Frynta et al. 2021), but the ranking of the stimuli according to self-reported fear is not necessarily affected. Thus, our ranking procedure, providing relative fear of each stimulus instead of absolute fear values, controls for this systematic effect.

## Conclusions

Snakes, scorpions, the centipede, and large carnivores were ranked by Somalis as the most frightening part of the local fauna. These fears seem biologically relevant since many species from these groups are either venomous or top predators, i.e., dangerous to humans. However, since some of these stimuli were, in fact, harmless to humans, we suspect that Somalis generalized their fear based on the group membership of the stimuli. Furthermore, the general pattern of fears is compatible with those previously reported from the WEIRD societies, with two especially noteworthy exceptions. First, lizards were ranked rather high—a probable result of culturally transmitted beliefs about their dangerousness. Second, the fear of spiders was reduced in favor of the fear elicited by another group of chelicerates—the scorpions. A follow-up study is needed to deepen our understanding of how self-reported fear relates to implicit measures of fear, and ultimately, what factors might be behind the patterns of fear observed in this study.

### Supplementary Information

Below is the link to the electronic supplementary material.

Supplementary file1 (XLSX 74.5 kb)

## Data Availability

All data generated or analyzed during this study are included in this published article in its Supplementary Information files (Online resources 1–3).
